# 慢性移植物抗宿主病一线药物治疗的真实世界研究

**DOI:** 10.3760/cma.j.cn121090-20250923-00435

**Published:** 2026-04

**Authors:** 晓倩 董, 云地 何, 世勤 黄, 筱淇 王, 瑞昊 黄, 曦 张

**Affiliations:** 1 陆军军医大学第二附属医院（新桥医院）血液病医学中心，血液生态与智慧细胞科学创新中心，解放军血液病中心、全军临床重点专科、重庆市临床重点专科，重庆 400037 Medical Center of Hematology, Xinqiao Hospital of Army Medical University, Chongqing Key Laboratory of Hematology and Microenvironment, State Key Laboratory of Trauma and Chemical Poisoning, Chongqing 400037, China; 2 金凤实验室，重庆 400037 Jinfeng Laboratory, Chongqing 400037, China

**Keywords:** 慢性移植物抗宿主病, 一线治疗, 疗效, 器官反应, 症状负荷, Chronic graft-versus-host disease, First-line treatment, Efficacy, Organ response, Symptom burden

## Abstract

**目的:**

系统评估慢性移植物抗宿主病（cGVHD）患者接受以糖皮质激素为基础的一线治疗的疗效、安全性、生活质量及相关影响因素，为优化治疗策略提供循证依据。

**方法:**

本研究为单中心回顾性真实世界研究，纳入2017年1月至2023年12月在陆军军医大学第二附属医院确诊cGVHD并接受一线治疗的243例患者。159例（65.4％）患者接受标准方案（激素联合/不联合钙调磷酸酶抑制剂），51例（21.0％）患者接受激素联合二线药物治疗方案（激素联合芦可替尼或西罗莫司），33例（13.6％）患者因禁忌证一线治疗未使用激素（单用芦可替尼）。主要终点为治疗6个月后的客观缓解率（ORR）和无失败生存（FFS）率，次要终点包括器官特异性反应、生存率、非复发死亡率（NRM）、复发率及生活质量［采用Lee症状学量表（LSS）、医学结局研究36项简短健康调查量表（SF‑36）、五水平五维健康量表（EQ‑5D‑5L）评估］。

**结果:**

6个月时132例患者一线治疗达完全或部分缓解，ORR为60.0％，FFS率为73.6％。皮肤、关节/筋膜等器官反应较好（ORR分别为66.4％、68.2％），肺受累反应率最低（30.0％）。中位随访43.9个月，5年总生存率为80.0％，治疗有效组显著高于治疗无效组（89.3％对75.4％，*P*<0.01）；5年NRM为7.5％，治疗有效组显著低于治疗无效组（3.6％对12.6％，*P*＝0.01）。多因素分析显示肝脏受累是一线治疗有效的危险因素（*OR*＝0.506，*P*＝0.021）。生活质量调查显示，治疗有效组LSS症状负荷显著低于无效组［（7.3±5.6）分对（12.3±7.8）分，*P*＝0.01］，而SF‑36躯体健康总评、精神健康总评及EQ‑5D‑5L量表效用指数差异无统计学意义。

**结论:**

以糖皮质激素为基础的一线治疗在cGVHD患者中显示出可接受的疗效并可以改善部分患者症状负荷，不同器官反应存在显著异质性，肝脏受累提示预后不良。

## Abstract (en)

**Objective:**

To systematically evaluate the efficacy, safety, quality of life, and related influencing factors of first-line treatment in patients with chronic graft-versus-host disease（cGVHD）and to provide evidence-based support for optimizing treatment strategies.

**Methods:**

This single-center retrospective analysis involved 243 patients diagnosed as having cGVHD who received first-line treatment at the Second Affiliated Hospital of Army Medical University between January 2017 and December 2023. Of the patients, 159（65.4％）received the standard regimen（corticosteroids with or without calcineurin inhibitors）, 51（21.0％）received corticosteroids plus a second-line agent（ruxolitinib or sirolimus）, and 33（13.6％）did not receive corticosteroids as first-line therapy due to contraindications（ruxolitinib alone）. The primary endpoints were the overall response rate（ORR）and failure-free survival（FFS）at 6 months after treatment. The secondary endpoints comprised organ-specific responses, survival rates, nonrelapse mortality（NRM）, recurrence rates, and quality of life［assessed using the Lee Symptom Scale（LSS）, the 36-Item Short Form Health Survey（SF-36）, and the EuroQol 5-Dimension 5-Level instrument（EQ-5D-5L）］.

**Results:**

At 6 months after treatment, 132 patients achieved complete or partial response to first-line therapy, yielding an ORR of 60％ and an FFS of 73.6％. ORRs were higher in the joint/fascia（68.2％）and the skin（66.4％）and the lowest in the lungs（30.0％）. The median follow-up was 43.9 months, the 5-year overall survival rate was 80.0%, and the 5-year NRM was 7.5％. Both overall survival and nonrelapse mortality were significantly better in responders than in nonresponders（*P*<0.01 and *P*＝0.01, respectively）. Multivariate analysis identified liver involvement as an independent risk factor for adverse treatment reactions（*OR*＝0.506, *P*＝0.021）. Quality-of-life surveys indicated that the symptom burden was significantly lower in responders than in nonresponders（7.3±5.6 *vs* 12.3±7.8, *P*＝0.01）, whereas no significant between-group differences were observed in SF-36 physical and mental component scores or the EQ-5D-5L utility index.

**Conclusion:**

Glucocorticoid-based first-line treatment showed acceptable efficacy and reduced symptom burden in some patients with cGVHD. However, significant heterogeneity was observed in treatment responses among different organs, and liver involvement indicated poor prognosis.

异基因造血干细胞移植（allogeneic hematopoietic stem cell transplantation, allo‑HSCT）是目前急性白血病等高危血液系统恶性肿瘤的重要根治手段，已在全球范围内广泛开展。然而移植物抗宿主病（graft‑versus‑host disease, GVHD）仍是限制患者长期生存获益的关键并发症之一。慢性GVHD（chronic GVHD, cGVHD）是allo‑HSCT最常见的远期免疫并发症之一，其累积发生率为30％～70％，具有显著的临床异质性，病程迁延且常伴持续性炎症及纤维化，严重者可造成多器官功能损害，显著影响患者长期生存和生活质量[1]–[3]。

随着移植技术和现代支持治疗手段的不断进步，allo‑HSCT受者生存期显著延长，cGVHD患者的长期规范化管理已成为当前移植后照护的核心目标。现行国内和国际专家共识均推荐糖皮质激素联合或不联合钙调磷酸酶抑制剂（calcineurin inhibitors, CNI）作为cGVHD一线标准治疗方案[4]–[5]，然而既往多项研究提示糖皮质激素单药的总客观缓解率（overall response rate, ORR）仅约50％，半数患者存在激素依赖或激素抵抗等情况，长期使用糖皮质激素还会诱发代谢紊乱、感染、骨质疏松及肌肉骨骼并发症等严重不良反应[6]。因此亟需真实世界大样本数据，全面评估当前一线治疗实际疗效、安全性及其对生活质量的影响，从而进一步识别影响糖皮质激素治疗反应和预后的关键临床特征，为后续优化个体化治疗策略和新型免疫调节药物奠定循证基础。

本研究回顾性分析我中心近年来确诊并接受一线系统治疗的cGVHD患者临床数据，系统评估总体及器官特异性治疗反应并探索潜在影响因素，旨在为提高cGVHD长期管理水平、改善患者预后和生活质量提供新证据。

## 病例与方法

一、患者来源

本研究为单中心回顾性真实世界研究，研究人群为2017年1月至2023年12月在陆军军医大学第二附属医院诊断为白血病行allo‑HSCT后发生cGVHD的患者。纳入标准：①行allo‑HSCT治疗白血病且移植后有完整随访数据；②自我报告存在活跃的cGVHD症状；③随访期间经门诊医师确诊为需要全身治疗的cGVHD并接受一线治疗。排除标准：①存在严重器官功能障碍；②临床上未控制的活动性感染；③具有严重的传染性疾病；④患者移植资料或随访数据缺失。

二、临床研究注册

本研究已在中国临床试验注册中心完成注册，并获得陆军军医大学第二附属医院医学伦理委员会批准（伦理批准号：2024-360-01）。研究严格遵循《赫尔辛基宣言》及其后续修订版本的伦理原则执行，所有患者均接受了标准的移植和随访治疗管理。鉴于本研究为非干预性回顾性分析，且所涉临床数据在分析前已进行匿名化处理，伦理委员会同意豁免获取书面知情同意。研究过程中已采取相应措施确保患者隐私和数据安全，符合国家和机构关于医学科研数据使用与管理的相关规定。

三、临床评估和定义

本研究的主要研究终点为一线cGVHD治疗方案启动6个月后患者的整体疾病缓解情况，根据美国国立卫生研究院（NIH）标准定义为完全或部分缓解，在一线治疗开始后3个月、6个月和12个月进行临床评估。如果在一线治疗开始后给予新的免疫抑制药物（immunosuppressive medicines, ISM），则停止临床评估。完全缓解（complete response，CR）定义为cGVHD的所有症状消退；部分缓解（partial response，PR）定义为至少一个器官等级的改善，其他器官没有cGVHD的进展；混合反应（mixed response，MR）定义为一个器官的改善，而其他器官进展；疾病进展（progressive disease, PD）定义为至少一个器官部位进展，其他部位没有任何改善；稳定的器官受累且分级没有任何变化被归类为疾病稳定（stable disease, SD）。无失败生存（failure free survival, FFS）定义为一线治疗期间没有使用进一步的ISM。总生存（overall survival，OS）定义为从确诊cGVHD并启动一线治疗到随访结束或患者死亡。非复发死亡定义为从确诊cGVHD并启动一线治疗后至随访截止，患者因除血液系统疾病复发以外的任何原因导致的死亡事件。累计复发定义为确诊cGVHD并启动一线治疗至随访结束，患者血液系统本病复发的事件。ORR和FFS率的计算基于意向治疗分析。如果患者没有完成12个月的整个随访期，则从尚未完成的第1个随访时间点（3个月、6个月或12个月）开始，相应的患者被排除在ORR和FFS率计算之外。若患者出现疾病复发或者死亡，相应患者被排除在ORR和FFS率计算之外。器官特异性缓解的评估以患者基线时cGVHD的器官受累严重程度作为参照。器官特异性缓解的基线评估在一线治疗开始时进行，并在3、6和12个月后重复评估其缓解情况。缺少临床评估的患者被归类为无改善。为了评估预测标志物，将患者分为治疗有效组（CR、PR）和治疗无效组（除达到CR、PR外的所有患者）。所有治疗反应均由专业的临床医师进行评估。随访期间，本研究通过向患者发放调查问卷，采用Lee症状学量表（LSS）评估症状负荷，医学结局研究36项简短健康调查量表（SF-36）、五水平五维健康量表（EQ-5D-5L）评估生活质量[7]。

四、统计学处理

所有统计分析均使用R 4.5.3完成。使用Kaplan-Meier曲线对OS进行分析，Log-Rank检验进行组间比较。对于非复发死亡率（NRM）和累计复发率，考虑存在竞争风险关系，采用Fine-Gray模型进行组间比较。使用Cox回归分析影响生存的相关潜在因素；使用Logistic回归分析影响治疗反应的相关潜在因素。采用标准算法计算量表得分，定量资料符合正态分布者用均值±标准差表示，计数资料以频数（构成比）表示。以*P*<0.05为差异有统计学意义。

## 结果

一、临床特征

本研究共纳入243例患者，其中男性147例，女性96例。cGVHD预防方案以临床常用抑制剂组合为主，即CNI+甲氨蝶呤+霉酚酸酯，部分患者加用抗胸腺细胞球蛋白（ATG）。cGVHD的中位发生时间为移植后5.4（2.8～26.6）个月。所有患者基线人口统计学及移植特征详见表1。

**表1:** 243例接受一线治疗的慢性移植物抗宿主病（cGVHD）患者临床特征

特征	数值
性别［例（％）］	
男性	147（60.5）
女性	96（39.5）
移植时年龄［岁，*M*（范围）］	31（2～60）
原发病［例（％）］	
急性髓系白血病	145（59.7）
急性淋巴细胞白血病	73（30.0）
慢性髓细胞性白血病	7（2.9）
其他白血病	18（7.4）
移植类型［例（％）］	
亲缘间全相合移植	68（28.0）
非亲缘间全相合移植	24（9.9）
非亲缘间微小不相合移植	26（10.7）
单倍体移植	125（51.4）
cGVHD发生与移植间隔［月，*M*（范围）］	5.4（2.8～26.6）
随访时间［月，*M*（范围）］	43.9（2.9～90.8）
aGVHD病史［例（％）］	47（19.3）
cGVHD严重程度（NIH分级）［例（％）］	
轻度	43（17.7）
中度	158（65.0）
重度	42（17.3）
cGVHD受累器官［例（％）］	
皮肤	152（62.6）
肝脏	138（56.8）
眼	69（28.4）
口腔	58（23.9）
胃肠道	28（11.5）
关节/筋膜	23（9.5）
肺	10（4.1）
cGVHD患者受累器官总数［例（％）］	
1	95（39.1）
2	79（32.5）
3	52（21.4）
4	15（6.2）
5	2（0.8）
一线治疗方案［例（％）］	
标准方案	159（65.4）
激素联合二线药物	51（21.0）
无激素方案	33（13.6）

**注** aGVHD：急性移植物抗宿主病；NIH：美国国立卫生研究院；标准方案：激素联合/不联合钙调磷酸酶抑制剂；激素联合二线药物：激素联合芦可替尼或西罗莫司；无激素方案：单用芦可替尼

在一线治疗开始时，42例（17.3％）患者出现重度cGVHD，158例（65.0％）出现中度cGVHD，另有43例（17.7％）轻度cGVHD患者，经临床医师评估后，开启一线治疗。cGVHD受累器官表现为皮肤（152例，62.6％）、肝脏（138例，56.8％）、眼（69例，28.4％）、口腔黏膜（58例，23.9％）、胃肠道（28例、11.5％）、关节/筋膜（23例，9.5％）以及肺（10例，4.1％）。经专业血液科临床医师评估后，159例（65.4％）患者接受标准方案（激素联合/不联合CNI），51例（21.0％）患者接受激素联合二线药物治疗方案（激素联合芦可替尼或西罗莫司），33例（13.6％）患者因禁忌证一线治疗未使用激素（单用芦可替尼）。

二、疗效分析

一线治疗6个月后，90例达到CR，42例达PR，13例MR，5例SD，12例PD。58例患者因PD使用额外的免疫抑制药物。有13例患者出现疾病复发（其中1例患者因复发死亡），3例患者因GVHD死亡，2例患者随访未达6个月，5例患者失访，共23例被排除在ORR、FFS率计算之外，因此随访6个月时，ORR为60.0％（132/220），FFS率为73.6％（162/220）（图1）；标准方案组、激素联合二线药物组及无激素方案组的ORR分别为63.2％、43.8％、71.4％，差异有统计学意义（*P*＝0.03）（图2）；不同受累器官特异性反应不同，ORR由高到低依次为：关节/筋膜（68.2％）、皮肤（66.4％）、胃肠道（56％）、口腔（55.6％）、肝脏（54.7％）、眼（53.0％），肺部受累的ORR最低，仅为30.0％（图3）。

**图1:**
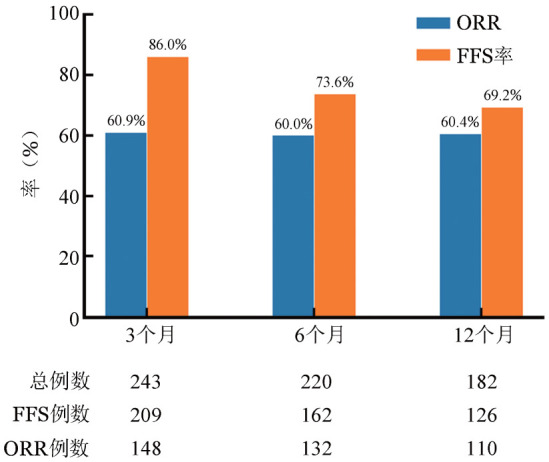
慢性移植物抗宿主病患者一线治疗开始后随时间推移的总体客观缓解率（ORR）和无失败生存（FFS）

**图2:**
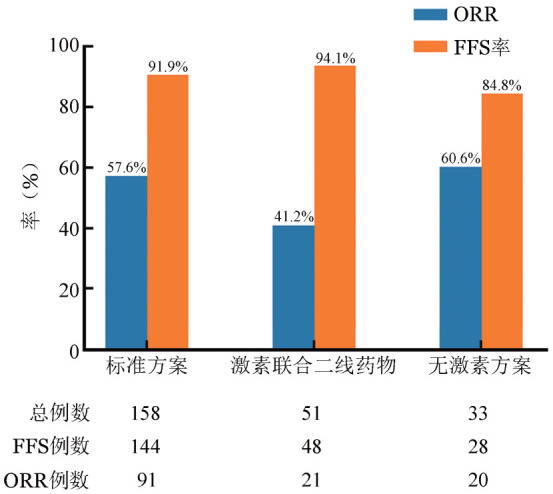
慢性移植物抗宿主病患者一线治疗6个月时不同一线治疗方案的客观缓解率（ORR）和无失败生存（FFS）

**图3:**
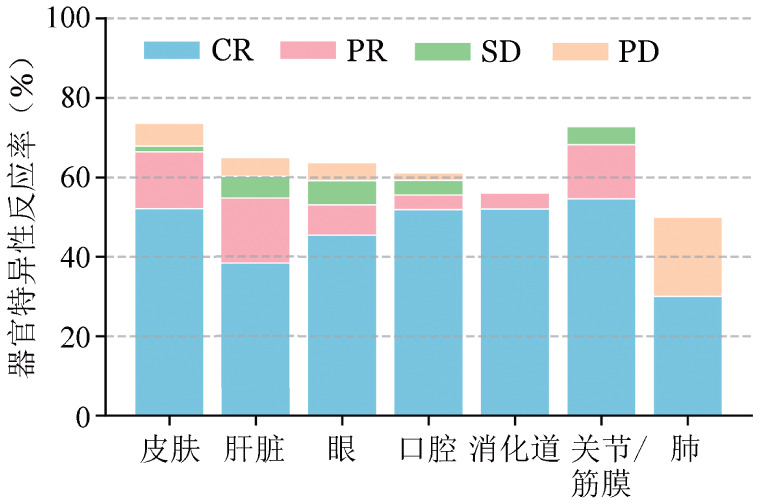
慢性移植物抗宿主病患者一线治疗6个月时器官特异性反应 **注** CR：完全缓解；PR：部分缓解；SD：疾病稳定；PD：疾病进展

三、生存分析

中位随访43.9（2.9～90.8）个月。截至末次随访，共33例患者出现本病复发，32例患者死亡，其中11例因复发死亡。所有患者5年OS率为80.0％（95％*CI*：70.1％～86.9％），治疗有效组和治疗无效组OS率（89.3％对75.4％）差异有统计学意义（*P*<0.01）。所有患者5年NRM为7.5％（95％*CI*：4.2％～12.0％），治疗有效组和治疗无效组NRM（3.6％对12.6％）差异有统计学意义（*P*＝0.01）；所有患者2年累计复发率为14.0％（95％*CI*：10.0％～19.6％），治疗有效组和治疗无效组累计复发率（10.0％对19.3％）差异无统计学意义（*P*＝0.11）（**扫描本文首页二维码查阅附图1**）。

四、回归分析

本研究对影响cGVHD患者OS的相关因素进行Cox回归分析（表2），将*P*≤0.5的变量代入多因素分析，结果显示一线治疗反应是影响cGVHD患者生存的独立保护因素（*HR*＝0.448，95％*CI*：0.209～0.959，*P*＝0.039）；对可能影响一线治疗反应的因素进行Logistic回归分析，将*P*≤0.2的变量代入多因素分析（表3），结果显示肝脏受累是一线治疗有效的危险因素（*OR*＝0.506，95％*CI*：0.283～0.903，*P*＝0.021）。肝脏受累患者和无肝脏受累患者的ORR分别为47.8％和61.9％，差异有统计学意义（*P*＝0.03）。

**表2:** 影响cGVHD患者总生存的预后因素Cox回归分析

变量	单因素分析		多因素分析	
	*HR*（95％*CI*）	*P*值	*HR*（95％*CI*）	*P*值
aGVHD病史（无/有）	0.833（0.342～2.025）	0.686		
cGVHD与移植间隔<5个月	0.930（0.457～1.893）	0.842		
供受者性别匹配（非女供男/女供男）	1.055（0.434～2.563）	0.907		
HLA 2个及以上点位不合	0.731（0.358～1.490）	0.388	0.806（0.373～1.743）	0.584
移植类型（亲缘间移植/非亲缘间移植）	0.920（0.377～2.249）	0.855		
cGVHD确诊时血清胆红素≥34.2 µmol/L	1.966（0.593～6.515）	0.269	1.834（0.522～6.442）	0.344
cGVHD确诊时外周血PLT<100×10^9^/L	0.909（0.416～1.986）	0.810		
cGVHD确诊时外周血ALC<1.0×10^9^/L	1.296（0.553～3.036）	0.551		
cGVHD确诊时外周血EOS<0.5×10^9^/L	1.175（0.355～3.887）	0.791		
GVHD相关因素				
皮肤排异（无/有）	1.316（0.634～2.732）	0.461	1.049（0.448～2.457）	0.913
眼排异（无/有）	1.312（0.631～2.726）	0.467	0.741（0.260～2.108）	0.574
口腔排异（无/有）	1.649（0.795～3.422）	0.179	0.704（0.237～2.094）	0.529
肝脏排异（无/有）	1.069（0.528～2.166）	0.853		
肺脏排异（无/有）	1.177（0.279～4.960）	0.825		
消化道排异（无/有）	1.343（0.516～3.497）	0.546		
关节/筋膜排异（无/有）	0.042（0.000～5.627）	0.204	–	–
cGVHD受累器官数				
<3个	参照			
3个	1.175（0.497～2.780）	0.714		
>3个	2.588（0.969～6.910）	0.058	4.987（0.726～34.275）	0.102
一线治疗反应（有效/失败）	0.419（0.205～0.858）	0.017	0.448（0.209～0.959）	0.039

**注** cGVHD：慢性移植物抗宿主病；aGVHD：急性移植物抗宿主病；HLA：人类白细胞抗原；ALC：淋巴细胞绝对计数；EOS：嗜酸性粒细胞计数。–：多因素完美分离，模型中剔除

**表3:** 影响cGVHD患者一线治疗反应的预后因素Logistic回归分析

变量	单因素分析		多因素分析	
	*OR*（95％*CI*）	*P*值	*OR*（95％*CI*）	*P*值
患者年龄（岁）				
<30岁	参照			
30～59岁	0.884（0.532～1.470）	0.635		
≥60岁	0.339（0.035～4.511）	0.457		
aGVHD病史（无/有）	1.073（0.566～2.035）	0.829		
cGVHD与移植间隔<5个月	0.988（0.591～1.653）	0.965		
供受者性别匹配（非女供男/女供男）	0.700（0.366～1.340）	0.282		
HLA匹配2个及以上点位不合	0.837（0.505～1.386）	0.489		
移植类型（亲缘间移植/非亲缘间移植）	1.688（0.888～3.210）	0.110	1.735（0.879～3.426）	0.112
cGVHD确诊时血清胆红素≥34.2 µmol/L	0.888（0.322～2.451）	0.819		
cGVHD确诊时外周血PLT<100×10^9^/L	1.270（0.724～2.228）	0.404		
cGVHD确诊时外周血ALC<1.0×10^9^/L	1.087（0.578～2.044）	0.759		
cGVHD确诊时外周血EOS<0.5×10^9^/L	1.953（0.893～4.273）	0.094	2.096（0.904～4.857）	0.085
GVHD相关因素				
皮肤排异（无/有）	1.323（0.783～2.235）	0.295		
眼排异（无/有）	0.655（0.374～1.147）	0.139	0.797（0.382～1.661）	0.545
口腔排异（无/有）	0.565（0.311～1.024）	0.060	0.789（0.356～1.752）	0.561
肝脏排异（无/有）	0.564（0.337～0.945）	0.030	0.506（0.283～0.903）	0.021
肺脏排异（无/有）	0.352（0.089～1.393）	0.137	0.332（0.073～1.510）	0.154
消化道排异（无/有）	0.712（0.323～1.569）	0.400		
关节/筋膜排异（无/有）	1.681（0.685～4.126）	0.257		
cGVHD严重程度（NIH分级）				
轻度	参照			
中度	0.564（0.280～1.135）	0.109	0.932（0.428～2.029）	0.860
重度	0.589（0.246～1.409）	0.234		
cGVHD受累器官数				
<3个	参照			
≥3个	0.364（0.205～0.649）	0.001	0.459（0.191～1.105）	0.082

**注** cGVHD：慢性移植物抗宿主病；aGVHD：急性移植物抗宿主病；HLA：人类白细胞抗原；ALC：淋巴细胞绝对计数；EOS：嗜酸性粒细胞计数；NIH：美国国立卫生研究院

五、生活质量评估

通过分析58份有效LSS量表、93份有效SF-36量表和93份有效EQ-5D-5L量表，治疗有效组和治疗无效组的LSS评估的症状负荷分别为（7.3±5.6）分和（12.3±7.8）分，差异具有统计学意义（*P*＝0.01）。治疗有效组和无效组的SF-36躯体健康总评（Physical Component Summary, PCS）分别为（43.3±11.3）分、（40.2±10.7）分，差异无统计学意义（*P*＝0.18）；精神健康总评（Mental Component Summary, MCS）分别为（40.4±9.3）分、（40.8±7.0）分，差异无统计学意义（*P*＝0.79）。治疗有效组和无效组的EQ-5D效用指数值分别为0.82±0.18、0.75±0.18，差异无统计学意义（*P*＝0.09）。

## 讨论

本研究回顾性分析了243例白血病患者在allo-HSCT后发生cGVHD并接受一线系统性治疗的真实世界数据，结果显示在以糖皮质激素为基础的一线治疗方案中，6个月ORR为60.0％，与既往研究报道相近[8]，支持糖皮质激素仍是目前cGVHD重要的初始治疗药物。

本研究发现各受累器官对一线治疗的响应存在显著异质性。其中，皮肤、关节/筋膜等组织反应率较高，提示其病理改变在免疫抑制干预下更易逆转；而肺脏反应率最低，仅为30.0％，与既往报道相符[9]，表明闭塞性细支气管炎/综合征等肺GVHD可能已呈不可逆纤维化改变，对现有免疫抑制策略敏感性不足，肝脏受累在本研究中被验证为一线治疗反应不佳的独立危险因素，提示对肝脏受累患者应使用更加积极的初始方案，必要时考虑早期联合新型免疫调节剂或个体化干预。

本中心患者5年OS率达80.0％，高于部分国际多中心回顾性研究数据[10]，可能与较为规范的诊治路径和及时干预有关。CR或PR患者的长期生存显著优于无反应者，表明早期、充分的疾病控制有助于改善远期生存。本研究中cGVHD治疗的反应状态与原发病复发之间未见显著差异，提示cGVHD治疗反应不能单独作为评估复发风险的判断标准，仍需结合微小残留病（MRD）监测及基础病风险进行综合管理。

本研究通过LSS、SF‑36及EQ‑5D‑5L量表对部分患者进行生活质量评估，结果显示治疗有效组LSS评分显著低于无效组，提示有效控制cGVHD不仅能改善器官反应，也能减轻症状负荷，但两组SF‑36躯体健康总评、精神健康总评及EQ‑5D‑5L量表效用指数差异未达统计学意义，可能与样本量有限、问卷回收率不足等因素有关，本研究结果表明以糖皮质激素为基础的一线治疗对症状控制直接获益，但在整体生活质量改善方面仍存在提升空间。

本研究还对不同一线方案（激素±CNI、激素联合二线药物及无激素治疗）的应用进行初步比较，这些结果为后续设计更高质量的前瞻性、随机对照研究提供数据基础。鉴于糖皮质激素长期应用的不良反应，国际研究正积极探索“激素+X”的早期联合治疗模式，如联合JAK抑制剂（Ruxolitinib）[11]–[12]或布鲁顿酪氨酸激酶抑制剂（Ibrutinib）[13]–[14]等，部分初步结果显示有望提高缓解率并减少激素用量，为未来cGVHD一线治疗策略的优化提供了新方向。

本研究也存在局限。作为一项单中心回顾性分析，其病例来源和治疗方案具有一定同质性；部分患者存在随访不全或器官评估缺失等问题，可能影响疗效评价的精确度。虽然本研究首次纳入了生活质量数据，但问卷回收率及样本规模有限，尚不足以全面反映生活质量变化。未来应开展多中心、前瞻性、长期随访的研究，结合生物标志物、影像学及动态生活质量监测进一步明确影响cGVHD治疗反应、生存及生活质量的关键因素，推动个体化和精准化管理的落地。

本研究通过分析真实世界样本数据同时首次结合生活质量评估量表，评估了以糖皮质激素为基础的一线治疗在cGVHD患者中疗效和器官反应的差异，指出肝脏受累提示预后不良，有效反应可显著改善症状负荷，为优化cGVHD一线治疗及提升患者综合获益提供循证依据。

## Supplementary Material


