# Supplementary figures and images for: First-line treatment for chronic graft-versus-host disease: a real-world study
Source: Zhonghua Xue Ye Xue Za Zhi. 2026 Apr;47(4):345–51. [Article in Chinese] doi: 10.3760/cma.j.cn121090-20250923-00435 (PMC13195549; doi:10.3760/cma.j.cn121090-20250923-00435)

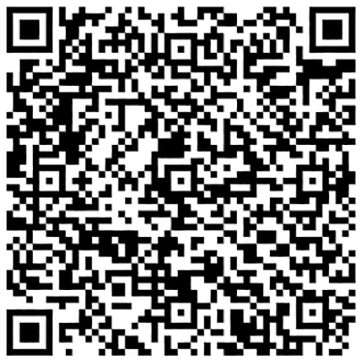

Supplement: Supplementary file 1 [file cjh-47-04-345-g004.tif]
